# Safe@Campus Virtual Reality Training for Campus Shooting Preparedness: Prototype Development and Usability Study

**DOI:** 10.2196/89471

**Published:** 2026-04-20

**Authors:** Jingzhen Yang, Hannah P Schneider, Krista K Wheeler, Lindsay Sullivan, Jason Wheeler, Brandon Abbott, David C Schwebel

**Affiliations:** 1 Center for Injury Research and Policy Abigail Wexner Research Institute Nationwide Children’s Hospital Columbus, OH United States; 2 Department of Pediatrics The Ohio State University Columbus, OH United States; 3 Division of Health Sciences School of Health and Rehabilitation Sciences, College of Medicine The Ohio State University Columbus, OH United States; 4 IT Research & Innovation Nationwide Children's Hospital Columbus, OH United States; 5 Department of Psychological and Brain Sciences The University of Iowa Iowa City, IA United States

**Keywords:** active shooter events, firearm violence, firearms, students, virtual reality

## Abstract

**Background:**

Campus shootings, though infrequent, result in significant loss of life, psychological trauma, and disruption to university communities. Traditional preparedness programs developed for K-12 settings do not translate well to university environments. Virtual reality (VR) offers an immersive and engaging method to enhance situational awareness and decision-making during high-stress events.

**Objective:**

This study aimed to develop the Safe@Campus prototype, a theory-informed, stakeholder-engaged VR-based prototype designed to prepare university students to recognize and respond to campus shooting threats, and to evaluate its initial usability and feasibility among undergraduate students.

**Methods:**

We followed a 2-phase, user-centered design process. Phase I (stakeholder-informed feasibility assessment and prototype refinement): through interviews with campus safety experts, firearm safety practitioners, school safety specialists, and students, we identified key content, scenario requirements, and implementation considerations. A 360-degree video–based VR prototype depicting an active shooter incident in a university classroom was developed using Unity3D, incorporating branching decision points aligned with the “run, hide, or fight” framework. Expert and user feedback guided iterative refinements. Phase II (student usability and acceptability testing): 2 focus groups with undergraduates at The Ohio State University (N=17) viewed a VR scenario and then participated in guided discussions about prior training experiences, the acceptability of VR, and recommendations for improvement. Transcripts were analyzed using constant comparative methods in ATLAS.ti (version 25).

**Results:**

The first focus group comprised 8 students (n=5, 63% female; n=3, 38% White, n=4, 50% Asian/Asian American), and the second comprised 9 students (n=6, 67% female; n=6, 67% White). Across both groups, 82% (14/17) reported participating in active shooter drills during K-12 schooling, yet many felt these experiences did not adequately prepare them for the complexity of university environments. The following four major themes emerged: (1) prior experience with active shooter drills: K-12 drills varied widely in realism and left students uncertain about appropriate actions in university settings; (2) need for university-specific training: participants noted substantial gaps in preparedness and expressed strong support for required, standardized training; (3) perceived usefulness of VR: students found VR highly engaging, realistic, and effective for reinforcing situational awareness and decision-making; and (4) recommendations for prototype improvement: students suggested increasing interactivity, adding time-pressured decisions, expanding scenarios to diverse campus spaces, and integrating the program into required university activities such as orientation.

**Conclusions:**

Safe@Campus is a feasible, acceptable, and engaging VR-based approach to campus shooting preparedness. Students viewed the immersive, decision-driven format as an effective way to build practical skills not addressed by traditional training. Future development should expand scenario diversity, increase interactivity, and evaluate program effectiveness in larger trials.

## Introduction

Firearm injuries kill over 48,000 people annually in the United States, equivalent to approximately 132 deaths per day [1]. Approximately 85,000 more individuals are shot and wounded annually [1]. Campus shootings represent a small but highly tragic and publicized portion of firearm injuries and deaths in the United States. Since the 2007 campus shooting at Virginia Tech, high-profile incidents at the University of Nevada, Las Vegas, and Michigan State in 2023 and, more recently, at Florida State University, the University of New Mexico, and Brown University in 2025, have heightened national concern [2-4]. Between 2013 and 2025, at least 418 incidents of gunfire occurred on university campuses across 44 states, resulting in 114 deaths and 312 serious injuries [5]. These events disrupt educational environments, strain emergency response systems, and generate lasting psychological trauma for survivors, bystanders, and surrounding communities [4,6-13]. Even indirect exposure to campus gun violence erodes feelings of safety, with recent surveys reporting that 65% of current and prospective college students worry about potential campus shootings [14-17].

Despite this widespread concern, few evidence-based, theory-informed interventions exist to help university students recognize, prevent, and respond to active shooter threats [18,19]. Active shooter drills are mandated in over 40 states and implemented in 95% of K-12 schools [20-24], but these programs have not been effectively adapted for use in university settings. One potential reason for the lack of adaptation is the nature of university campuses, which differ significantly from typical K-12 settings. University campuses are generally more open, cover larger geographic areas, contain diverse and sometimes nonlockable facilities, and serve a more independent adult population than K-12 settings [25]. Given the prevalence and profound impact of campus shooting events, there is a critical need to develop an effective firearm injury prevention program for use in university settings.

Virtual reality (VR) offers a promising strategy for such firearm injury prevention programs. VR simulations provide immersive, engaging, and interactive environments that enhance learning, accelerate skill acquisition, and increase knowledge retention [26-28]. VR has been widely used in education and injury prevention [29-31], including in law enforcement training for active shooter response [32,33]. It can help students recognize concerning situations and behaviors and reinforce the best ways to report possible threats [25]. Using this evidence, we developed and evaluated a prototype of Safe@Campus, a VR-based intervention. Unlike traditional K-12 active shooter drills, the fully-developed Safe@Campus program will address both the prevention of and response to campus shootings by providing guidance on how to recognize behavioral warning signs and report potential threats before violence occurs, as well as practical strategies for responding effectively during an active shooter incident [34-38]. Through immersive VR scenarios, students will practice systematic threat observation to recognize potentially dangerous environments, situations, and individuals. They will also rehearse rapid, critical decision-making based on the “run, hide, or fight” framework [39] and build confidence to act under stress, skills that extend beyond campus and can be applied in other public settings.

The aims of this study were to develop a prototype of the Safe@Campus program, a theory-informed, stakeholder-engaged VR-based prototype designed to prepare university students to recognize and respond to campus shooting threats, and to evaluate its initial usability and feasibility among undergraduate students. As an early step in this line of research, this study focuses on the development and usability testing of a single VR prototype scenario rather than an evaluation of effectiveness. These findings provide the foundation for future studies, including randomized clinical trials, to rigorously assess the intervention’s impact on behavioral and preparedness outcomes.

## Methods

### Theoretical Framework and Conceptual Model of Safe@Campus VR Intervention

Safe@Campus is a semi-immersive VR educational app that immerses users in realistic, risk-adjusted settings such as dormitory, classroom, and outdoor campus area environments, with the goal of enhancing situational awareness and response skills. Proximity-based decision-making and stress inoculation are emphasized to help students build coping skills and confidence in high-stress situations. All virtual stimuli are delivered through a commercially available VR headset (Pico Neo 3 Eye), providing an immersive 360-degree video experience with synchronized spatial audio.

Safe@Campus is underpinned by the theoretical constructs of both the experiential learning model [40-42], which emphasizes “learning by doing,” and the theory of planned behavior (TPB) [43,44], which highlights the role of intention in performing behaviors.

**Figure 1:**
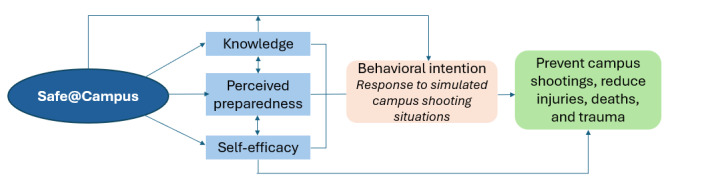
Conceptual model.

Conceptually, Safe@Campus aims to enhance university students’ ability to recognize, report, and respond to campus shooting threats, boost their self-efficacy to engage in these actions, and increase their perceived preparedness. Improvements in these abilities are hypothesized to lead to safer behavioral response intentions in simulated campus shooting situations, preventing future campus shootings, and reducing associated injuries and deaths (Figure 1).

### Development of the Safe@Campus VR Prototype

#### Platform Selection and Technical Implementation

VR training experiences are typically created in one of two ways. The first involves building a digital environment where users can navigate and interact, similar to a fully immersive video game. The second uses 360-degree video captured with a specialized camera, allowing users to view scenarios unfolding in real-world settings. Although 360-degree video offers fewer options for interactive elements than some alternatives, it presents several key advantages: it is significantly more cost-effective to produce, provides a consistent experience across all users, and places users in familiar environments with a high degree of realism [45].

The prototype tested in this study used a VR headset to immerse users in a 360-degree video environment, allowing them to rotate their bodies and view the scene from any direction. The scene unfolds around the user, guided by dialogue from an on-screen actor. While the companion’s lines are delivered verbally, often while making direct eye contact with the camera, the user’s responses are presented as scripted dialog boxes.

The prototype was developed in the Unity3D game engine, which enabled the creation of interactive interface elements prompting users to make decisions. These decisions impact the outcome of the scene. Multiple videotapes were filmed to account for the branching of the scenario. For example, if a user chose to hide, a corresponding video sequence showed the actor asking where to hide next. The user was then prompted to find a suitable hiding place from highlighted options around the room, and the actor responded to each choice by explaining why it was appropriate or inadequate.

#### Scenario Design and Instructional Structure

While the full Safe@Campus intervention is designed to address both the prevention of and response to campus shooting situations, the prototype evaluated in this study consisted of a single scenario focused specifically on response during an active shooter incident in a classroom building setting. The experience begins with the immersed user and their virtual companion leaving a classroom, casually discussing a difficult exam, and making plans to meet friends for tacos. This light, relatable interaction is abruptly interrupted by the sound of gunshots. Initially, both characters are unsure whether the noises were gunfire or something less serious. However, moments later, their phones buzz with an emergency alert, confirming that an active shooter is present on campus. The atmosphere quickly shifts from light and relatable dialogue to background noises filled with the sounds of panic, yelling, and running. At this point, the user is prompted by the program to make a critical decision: run, hide, or fight. Each selection leads to a short scenario illustrating key safety strategies and lessons:

- Run: the user evacuates using the stairs, leaving their belongings behind.
- Hide: the user seeks secure cover nearby, in a janitor’s closet.
- Fight: the program discourages this, with the companion advising against it.

The simulation concludes with an expert message reinforcing that running is the safest and preferred option, hiding is a viable secondary option, and fighting should be considered only as a last resort. In this early-stage prototype, prompts and highlighted options were intentionally included to reinforce the “run, hide, or fight” framework. Future iterations may introduce more advanced, open-ended scenarios that better reflect real-world conditions and require users to identify response options independently.

### User-Centered Development: Feasibility and Usability Test of the Safe@Campus Prototype

**Figure 2:**
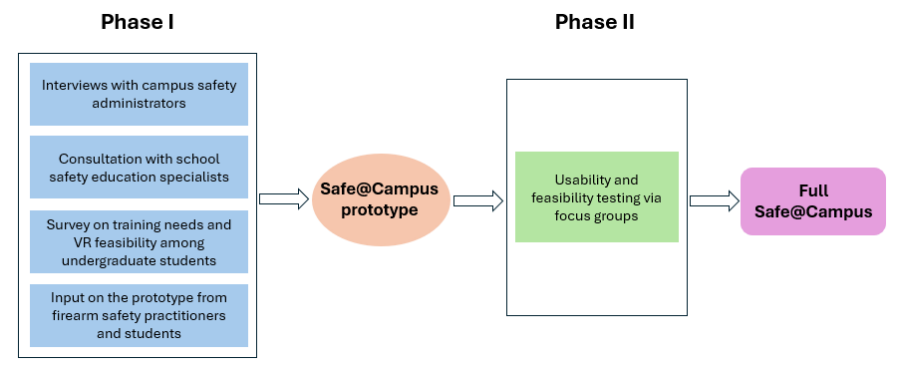
Process of developing and testing the Safe@Campus prototype. VR: virtual reality.

The Safe@Campus prototype was refined and tested through a collaborative, user-centered design process. As outlined below and shown in Figure 2, the refinement and testing occurred in two phases: (1) stakeholder-informed feasibility assessment and prototype refinement, and (2) student usability and acceptability testing.

#### Phase I: Stakeholder-Informed Feasibility Assessment and Prototype Refinement

##### Overview

Phase I assessed the feasibility of the Safe@Campus prototype and refined its design through structured input from key stakeholders. We consulted experts in digital health design, campus safety education, firearm safety research, and campus safety administration and law enforcement, as well as university students. These interviews had three primary objectives: (1) assess the prototype’s appropriateness for college students and its relevance to campus firearm safety; (2) gather stakeholder recommendations on visual and sensory design, content, and scenarios; and (3) obtain guidance on optimal implementation, including when, where, how, and under what conditions the intervention should be delivered. End users were engaged early and throughout the refinement process to ensure the prototype’s relevance, realism, and practical feasibility.

##### Interviews With Campus Safety Leaders and Law Enforcement

In February 2024, we conducted individual interviews with campus safety experts, including leaders from public safety at The Ohio State University (OSU), the University of Alabama at Birmingham, and the Regional Gun Violence Research Consortium in New York State, as well as a detective from the OSU Police Department specializing in campus shooting threats and safety education. These interviews explored the national landscape of campus emergency preparedness, opportunities for institutional partnerships, design considerations for VR-based training, the effectiveness and unintended consequences of existing active shooter preparedness programs, and the specific needs of college students. Experts emphasized that training should prepare students to respond not only on campus but also in public settings such as concert venues and shopping malls, underscoring the importance of practical self-defense and transferable skills across environments and scenarios. They also endorsed VR as an engaging and effective medium for student learning. Collectively, these consultations highlighted key programmatic gaps, institutional needs, and opportunities for collaboration.

##### Consultation With School Safety Education Specialists

In August 2024, we met with the leadership team and school safety education specialists from the Ohio School Safety Center (OSSC), including the director and the school policy and compliance manager. Established in 2019, OSSC employs 53 staff who support firearm safety training across both K-12 and higher education institutions. As part of our effort to gather feedback on the Safe@Campus prototype, we sought OSSC staff’s insights from their experience in campus firearm safety education and discussed the potential of VR to enhance situational awareness and decision-making during emergencies. They recommended tailoring “run, hide, or fight” scenarios to the unique features of university campuses, such as open layouts, diverse environments, and a student population more mature than at K-12 schools. Staff also noted that funding for firearm safety initiatives is more limited in higher education than in K-12 settings, highlighting the need for additional resources.

##### Undergraduate Survey on Training Needs and VR Feasibility

To assess student perspectives on training needs and VR feasibility, we conducted an anonymous online survey among undergraduates enrolled in a health sciences course at OSU. Among 45 respondents, 87% (n=39) rated active shooter training as “very” or “extremely important,” and 87% (n=39) considered VR-based training “feasible” or “very feasible.” These results demonstrate strong student support for VR as an engaging and acceptable approach to campus safety training.

##### Prototype Feedback From Firearm Safety Practitioners and Students

Based on initial feedback, we refined scripts and scenarios, recruited an actor, and filmed 360-degree videos for a Safe@Campus prototype. After multiple rounds of internal testing and refinement of the prototype, we conducted a focus group to gather information about the prototype with OSU campus police officers and 8 OSU student interns specializing in campus safety. Participants reviewed the prototype, evaluating its features, active shooter simulations, and scenario design. They reflected on their user experience, including what they “liked most” and “liked least.” A trained qualitative researcher facilitated the discussions.

Key recommendations for the prototype included (1) incorporating quicker decision-making options, (2) gradually escalating threat levels with appropriate trigger warnings, (3) ensuring institutional buy-in and coordination, and (4) expanding the intended audience to include faculty, staff, and administrators. These insights provided critical guidance for refining the Safe@Campus prototype to enhance its relevance, usability, and institutional feasibility. We incorporated this feedback into the final prototype version prepared for Phase II usability and feasibility testing.

#### Phase II: Student Usability Testing of the Safe@Campus Prototype

Phase II aimed to evaluate the usability and acceptability of the Safe@Campus prototype through 2 focus groups with OSU undergraduate students.

#### Focus Group Guide

The focus group guide was developed based on the theoretical frameworks of the experiential learning model [40-42], the TPB [43,44], and existing literature on campus shooting prevention and response [15-18,36,46,47]. It was pilot tested and refined based on feedback from the research team and external collaborators, including firearm safety experts and qualitative researchers.

For this study, we concentrated on questions related to student perspectives and experiences in four areas: (1) prior active shooter training (eg, “What personal experience do you have with active shooter training? When and where did you have the experience?”), (2) the need for active shooter training for college students (eg, “What are your thoughts about conducting active shooter training in college campus or among college students?”), (3) the usefulness of the VR-based training approach and feedback on the Safe@Campus prototype (eg, “Do you feel like it is an important issue in college campus? Why or why not?”), and (4) facilitators and barriers to implementing VR active shooter safety training in university settings (eg, “What barriers might prevent us from developing the VR active shooter safety training for college students?”).

#### Participants

Participants were undergraduate students. Eligible students were aged 18-25 years and enrolled at the OSU Columbus campus, English-speaking, and provided informed consent.

#### Study Procedure

A convenience sample of undergraduate students from OSU was recruited for 2 focus groups held in an OSU classroom in October and November 2024. Study information was shared with potential participants through word of mouth. Interested students contacted the research coordinator to be screened for eligibility and, if eligible, to schedule a focus group. Two focus groups were scheduled and conducted. At the start of each session, a trained researcher (HPS) reviewed the study purpose and procedure, answered questions, and then obtained written consent from participants.

Each student then participated in a single in-person session with 8-10 participants (n=8 in Group 1 and n=9 in Group 2). After brief introductions and safety instructions, including a content warning and a reminder that students could stop participation at any time due to motion sickness, emotional discomfort, or any other reason, students independently engaged in the VR Safe@Campus prototype. The simulation placed users in a university classroom scenario that was disrupted by the sound of gunfire and a campus emergency alert indicating an active shooter. Participants were prompted to choose whether to run, hide, or fight, with each option illustrating key safety strategies. The simulation concluded with a police officer reinforcing the “run, hide, or fight” framework.

Following the VR experience, students participated in a semistructured focus group facilitated by 2 experienced qualitative social science researchers (HPS and LS) trained on the study protocol. A discussion guide was used to ensure consistency across groups while allowing flexibility to probe emerging themes. Participants were asked about their prior active shooter training, perspectives on implementing such training in university settings, and reactions to VR as a training tool. They also provided feedback on the prototype’s storyline, tone, dialogue, and character representation. Each focus group lasted 30-45 minutes. Sessions were audio-recorded with participant consent and transcribed verbatim by an institutional review board–approved transcription service for analysis.

#### Data Analysis

Transcribed focus group data were analyzed using the constant comparative method [48,49] through an iterative and systematic process. Two research team members (HPS and JY) independently read each transcript in full to familiarize themselves with the data and document initial impressions. Using an inductive approach, they conducted line-by-line coding of meaningful text segments, generating open codes grounded in participants’ language. Codes were continuously compared within and across transcripts to identify similarities, differences, and recurring concepts, and were refined as analysis progressed. Related codes were grouped into broader categories and synthesized into higher-order themes and subthemes. Both categorizing and connecting strategies were used to organize themes by shared meaning and examine relationships across themes, contexts, and focus groups. ATLAS.ti (version 25; ATLAS.ti Scientific Software Development GmbH) was used to manage transcripts and coding. To enhance rigor, both coders independently coded all transcripts and met regularly to reconcile discrepancies through discussion and consensus with the broader research team. Analytic decisions and codebook revisions were documented throughout the process to ensure transparency and consistency.

### Ethical Considerations

All aspects of the study were approved by the institutional review board at Nationwide Children’s Hospital (IRB STUDY00004155). Written informed consent was obtained from each student participant prior to the study procedures. All participant data were deidentified to ensure the privacy and anonymity of participants. Participants were compensated with a US $20 ClinCard (Greenphire Inc) upon completion of the focus group.

## Results

### Participant Demographics

The first group comprised 8 participants (n=5, 63% female, n=3, 38% White, n=4, 50% Asian or Asian American, n=6, 75% junior- or senior-year students), and the second group comprised 9 participants (n=6, 67% female, n=6, 67% White, n=7, 88% junior- or senior-year students). More than 82% (14/17) reported having participated in active shooter drills during their K-12 education. All students had reviewed a required active shooter video training as part of the new student university orientation.

### Themes and Subthemes

#### Overview

The following four major themes and relevant subthemes were identified, as presented in Textbox 1 along with associated quotations: (1) prior experience with active shooter drills—defined as students’ past exposure to K-12 drills and related training activities; (2) need for university-specific training—which referred to students’ perceived gaps between K-12 preparation and the unique risks and demands of a large, diverse campus environment; (3) perceived usefulness of VR*—*representing students’ perceptions of VR as an immersive tool for situational awareness and decision-making; and (4) recommendations for prototype improvement—which referred to students’ suggestions to improve the VR training content, scenarios, and how to integrate the intervention into required university programs. Theme 4 comprised 3 subthemes: VR content, VR scenarios and design, and VR delivery.

**Theme, subtheme, quotation, and data source.**

**Theme 1. Prior experience with active shooter drills**

- “Most of the time, we just barricaded our classroom to just prepare. In some cases, depending on where the expected shooter was, we would evacuate the school in its entirety, but in other cases, we would just barricade up.” (Group 1)
- “It was called a lockdown drill, and it was probably three or four times a year.... I did it in elementary school through high school, but we would act it out. We’d sit really, really quiet and all the lights would go out and we’d have to hide in the corner away from the door and then someone would walk around and make sure they couldn’t see people and then if they could see people, they’d send an email and make sure that they fixed it.” (Group 1)
- “We did a lot of barricade drills where we’d have to put stuff in front of the door, and then they would try and open it, and then if they couldn’t open it, then we passed. But then, if they could open it, then it was a failure, and then the class had to talk about what to do better. And then I know some teachers had weapons that they could use or tell us where they were. It was not a real weapon, but just stuff that we could use to throw, and then they would tell us where that was.” (Group 2)
- “In high school, they had it very realistic...they would just make an announcement over the PA system and say where it was located. And then based off that, the teachers were supposed to react and have a plan set in place, whether it was run, hide, or fight. And then also, I know the teachers had a separate training day for active shootings outside of when students were in the building.” (Group 2)

**Theme 2. Need for university-specific training**

- “I feel like it’s needed because I remember thinking a few months ago when there was a shooting...if we’re in class and it happens, I’m just thinking does everyone know what to do? ...some of us are also like you don’t remember every single protocol and I just feel like it should be a mandatory thing in college too because if anything a shooter would have more free rein and more I feel like could go on rather than in a high school in one location.” (Group 1)
- “After coming to [university], there’s been multiple shooters that actually have been on campus. And honestly, I have no idea what to do other than run. And we’ve never actually been through a professional procedure, so I feel like it would be good if we did.” (Group 1)
- “I think they definitely need to prepare. In my perspective, coming from a really small high school, I never felt like in K-12 that an active shooter situation was a direct risk. But coming to [university], I feel like there have been shootings nearby or I’ve gotten an alert on my phone about people on campus with guns before. So, I feel like the preparation that I had in K-12 doesn’t carry over to college, and it does not prepare me for an active shooter situation in college.” (Group 2)
- “I feel like that’s a good thing to implement, but it’s also hard that you don’t want your students to know always 100% what your active shooter procedure may be just because if your shooter is a student then it’s like well then they know what you’re going to do and that aspect of making sure that everyone can still stay safe. But I think it is important that everyone should be aware in the scenario, like, okay, we do need to lock down the area, and instead of just feeling like you’re trapped, like no, they’re doing something here and need to figure it out.” (Group 2)

**Theme 3. Perceived usefulness of Virtual Reality (VR)**

- “I really thought it brought out situational awareness, and it made you feel like in the place at that moment. Like, what would you actually do? And I really liked how there were multiple exits, so you got to choose, and it would correct you and tell you if your decisions were right or wrong because this could be a real scenario as well. You don’t want to make the wrong decision when it actually happens.” (Group 1)
- “I feel like VR is a unique way.... Because it’s made to make you feel like you’re in that situation. So, I’ve never done anything like that, but it was cool to actually have to look around the room, think about nearest, furthest exits. Like, actually hear where the gunshots are in relation to you.” (Group 1)
- “It’s good to get a visualization of what that situation would be like without having to, like we said in our elementary schools and high schools and stuff, they actually went through and had everyone do it, but in college, there was not really an opportunity for that.” (Group 2)
- “I ran through all three scenarios of the run, fight, and hide, and I liked that after everyone, it explained why this was the best option, or fighting, it was like this is your worst case, don’t ever do this. And I think it’s good [it was] explaining why that was the correct choice just so that if you’re ever in a stressful situation, you can kind of break it down a little bit and be like, okay, well I know this, and then I can kind of think through it a little bit more logically.” (Group 2)

**Theme 4. Recommendations for prototype improvement**

- Subtheme 4.1: VR content
  “People who were feeling different things. The girl we’re interacting with was kind of calm in the situation, and I wouldn’t expect it. I think the only thing she did was lower her voice after the shooting. Probably having other people who react differently.” (Group 1)
  “I feel like there should also be a timer associated with your decisions to have more chaos. Because in a real event, you won’t actually have infinite time to choose. I feel like having a time rule like, oh I have this set of time, I have to make an option right now.’ And then that’ll also train people to make the right decision when that actually does happen.” (Group 1)
  “I would’ve liked to see more options as far as where to hide or where to run, and just to visualize why the option I chose is incorrect instead of having it be explained. I think being able to see it for yourself would also be more helpful.” (Group 2)
  “I feel like it’d be nice if they had someone telling you this, ...a campus resource officer or something like that, or a police officer saying think this was the best option, good job, or that was the worst option.” (Group 2)
- Subtheme 4.2: VR scenarios and design
  “I think a lot of the training focuses on indoors. Maybe if you’re walking out of the student union, you’re like wait, there’s a huge lawn space. If I’m running, I’m going to be seen, where would I go? I think outside is a whole different thing.” (Group 1)
  “Just like the areas like the student union. It could be literally in the student union, where are your exits, where can you go.” (Group 1)
  “The library.” (Group 2)
  “I feel like a dining hall.” (Group 2)
- Subtheme 4.3: VR delivery setting
  “Maybe you would have the option if you’re a freshman, you would take it in that [freshmen] class because it’s in class, people must go to it. And then for a transfer when they have their orientation to do it.” (Group 1)
  “If you’re at the student union or something a day and you go and if you put this VR thing on.... If you’re an outside, it’d probably work better. Then you get free food.” (Group 1)
  “I would say it should be something that could be a grade for a student or something implemented at orientation that you kind of can’t avoid because personally I’m not going to go out of my way in the student union to do an active shooter thing.” (Group 2)
  “Most students are required to live in a dorm their first and second year, [so] making it a part of an RA task and looking at it, and every month you could do a different floor of the dorm.... So that way it still keeps it in a small group.” (Group 2)

#### Theme 1: Prior Experience With Active Shooter Drills

Participants recalled extensive exposure to active shooter drills in K-12 schools, most of which emphasized lockdowns, barricades, and real-world simulated scenarios. While these experiences fostered familiarity with basic safety actions, the approaches varied in realism and thoroughness. Some recalled routine lockdowns in which “we’d sit really, really quiet and all the lights would go out and we’d have to hide in the corner away from the door” (Group 1). Others described more interactive drills that evaluated their responses, such as “put[ting] stuff in front of the door...and then if they couldn’t open it, we passed” (Group 2). Several remembered creative training formats, like a video with an “Alice in Wonderland” theme where letters stood for different actions, or teachers pointing out improvised objects to throw if necessary. Still, the depth of practice ranged; while some schools practiced evacuations, others focused exclusively on barricading. This left participants with mixed impressions of preparedness.

#### Theme 2: Need for University-Specific Training

Students emphasized that earlier training did not adequately prepare them for the risks they now face on a large university campus. Many noted that while their K-12 environments felt relatively secure, universities posed different challenges, such as larger, open campuses and a multitude of diverse buildings. One participant reflected, “Coming to [university], I feel like there have been shootings nearby...the preparation I had in K through 12 doesn’t carry over to college” (Group 2). Others stressed the importance of coordinated strategies, given the diverse backgrounds and experiences of the student body: “Everybody comes from so many different places...it’ll be important just to implement some kind of strategy” (Group 2). Students also raised concerns about the lack of professional, standardized training, with 1 admitting, “After coming [to university], there’s been multiple shooters...and honestly I have no idea what to do other than run” (Group 1). Some even worried about balancing safety with security, noting that while procedures should be clear, they must also be designed so that potential shooters who are students themselves cannot exploit the knowledge of planned responses.

#### Theme 3: Perceived Usefulness of VR

Students viewed 360-degree video VR training as a highly engaging and memorable approach, noting that this approach was preferred over traditional videos or lectures. Many valued how VR immersed them in realistic scenarios and required them to engage and make active decisions. One participant shared, “I really thought it brought out situational awareness, and it made you feel like [you were] in the place at that moment” (Group 1). Others appreciated the ability to learn through reinforcement; 1 noted, “I liked that after every scenario it explained why this was the best option...so that if you’re ever in a stressful situation you can think through it more logically” (Group 2). For some, the novelty of VR heightened its impact: “I’ve only used VR three times in my life, so it’s definitely more impactful. I’d definitely pick up some cues if it were to happen from the VR” (Group 1). Overall, students felt that VR helped bridge the gap between abstract preparation and practical readiness.

#### Theme 4: Recommendations for Prototype Improvement

This theme comprised 3 subthemes: VR content, VR scenarios and design, and VR delivery setting.

#### Subtheme 4.1: VR Content

While VR was seen as effective, students called for greater interactivity and realism to strengthen its training value. They wanted to “see more options as far as where to hide or run” (Group 2) and even visualize why certain choices were incorrect. Others suggested sensory enhancements, such as louder sound, vibrations, or simulated chaos to mirror the stress of real events: “I want to feel like vibrations...if there were more people in the room then it’d be more immersing” (Group 2). The addition of timed choices was another common request, with 1 participant explaining, “In a real event, you won’t actually have infinite time to choose...[a timer] will also train people to make the right decision when that actually does happen” (Group 1).

#### Subtheme 4.2: VR Scenarios and Design

Students suggested that VR scenarios should extend beyond classrooms to encompass a wider range of campus-specific spaces they encounter in their daily routines. Locations like “the student union...the library...[and] a dining hall” (Group 2) were highlighted as critical areas where preparedness would be just as important as in classrooms. They also emphasized the need to incorporate outdoor environments, noting that most current training focuses on indoor settings: “Maybe if you are walking out of the student union, there’s a huge lawn space. If I’m running, I’m going to be seen, where would I go? Outside is a whole different thing” (Group 1). Students felt that practicing in realistic-feeling, recognizable simulated spaces, both indoors and outdoors, would make the training more relevant and effective.

#### Subtheme 4.3: VR Delivery Setting

Finally, students emphasized the importance of how the training is delivered and who participates. They felt voluntary participation would be inadequate and recommended integrating VR training into university-required programs such as orientations, first-year courses, or residence life activities. One student explained, “requiring at orientation...because there was a lot of incoming freshmen where we just kind of did nothing with their peer leader.” (Group 1), while another added*,* “If it is required, more students would get it done” (Group 2). Others suggested enforcement strategies such as course requirements, account holds, or resident assistant led dorm sessions. Overall, students viewed embedding VR training within existing academic curriculum or residential structures as the most effective way to ensure participation and maximize its impact.

## Discussion

### Brief Summary of Main Findings Relative to Study Aims

This study describes the development of Safe@Campus, a theory-informed, stakeholder-engaged VR-based program designed to prepare university students to recognize and respond to campus shooting threats. Specifically, the study evaluated the initial usability and feasibility of a Safe@Campus prototype among undergraduate students. Consistent with our aims, findings indicate that the prototype is feasible to implement and highly acceptable to students. Although most participants had prior K-12 active shooter training, they reported feeling unprepared for the scale and complexity of university settings. Students perceived VR-based training as immersive and more engaging than traditional video- or lecture-based approaches. They also offered specific recommendations to enhance realism, interactivity, and campus relevance, directly informing the next phase of refinement and evaluation. The results support continued development of Safe@Campus and evaluation of the program in continuing research studies, including randomized clinical trials.

### Interpretation of Findings, Implications, and Comparison to Existing Literature

#### Gaps in Preparedness From K-12 to University Settings

A key finding was a clear disconnect between prior K-12 active shooter drills and students' perceived readiness in university settings. This perception aligns with prior research demonstrating that preparedness strategies developed for K-12 settings do not seamlessly translate into higher-education environments [10,50,51]. University campuses are larger, more open, and more diverse in building types and populations, making standardized lockdown procedures and coordinated response more complex [25,38,47,52]. The students are older, more mature, and more independent. Our findings provide qualitative evidence that drills focused on confined classroom settings may not prepare students to navigate the spatial and organizational complexity of university campuses. Participants also emphasized the need for coordinated and standardized training that reaches the entire student body, reflecting recommendations for institution-wide prevention and response frameworks in higher education [10,38,52]. Moreover, students highlighted the challenges of balancing transparency with security, recognizing that while clear procedures are necessary, overly detailed protocols could potentially be exploited. This tension mirrors concerns raised in the campus threat management literature [36,47,53].

#### Experiential Learning and the Role of VR

Students consistently described VR as immersive, memorable, and more engaging than traditional didactic formats. These findings concord with experiential learning theory, which emphasizes active engagement and reflection as drivers of skill acquisition [40-42]. The Safe@Campus prototype required participants to assess environmental cues, interpret auditory signals, and make rapid decisions under simulated stress, elements that align with experiential learning principles and the TPB framework guiding the intervention [43,44]. Previous evidence supports the effectiveness of VR for enhancing knowledge retention and situational awareness across educational and safety contexts [26,31,33,54,55], including law enforcement and emergency response training [32,33]. Our findings suggest similar potential for university firearm injury prevention. Participants particularly valued the structured feedback explaining why specific choices aligned, or did not align, with the “run, hide, or fight” framework [39]. Such feedback may strengthen perceived behavioral control and self-efficacy, key constructs within TPB [43,44].

#### Integrating Prevention and Response Within a Public Health Framework

The Safe@Campus prototype evaluated in this study focused on response to an active shooter incident. When fully developed, Safe@Campus is conceptually designed to integrate prevention (recognition and reporting of warning signs) along with response strategies. This approach aligns with public health models emphasizing upstream identification of warning behaviors alongside crisis response planning [34-38,46]. Research on leakage warning behaviors and threat assessment underscores the importance of empowering individuals to recognize and report concerning behaviors before violence occurs [36,37]. By combining recognition, reporting, and response within an immersive platform, the conceptualized Safe@Campus aligns with comprehensive campus violence prevention frameworks [10,38,52].

#### Implementation and Policy Implications

Students strongly supported embedding VR training within required university programming, such as orientation, first-year seminars, or residence life activities. This recommendation is consistent with existing research suggesting that campus safety initiatives are most effective when standardized, required, and institutionally integrated rather than voluntary [10,38,52]. Given persistent student concern about campus safety [14-17], scalable and engaging training models may help address both student preparedness and perceived safety.

Compared with resource-intensive live drills, VR-based programs may offer a more feasible and potentially cost-effective alternative, particularly in university settings where funding for firearm safety initiatives is limited [56]. Partnerships among universities, campus police, state school safety centers, and public health agencies may enhance sustainable implementation.

An important consideration for broader dissemination is generalizability across campuses with different physical layouts and resources. The Safe@Campus prototype reflects a typical university classroom setting that students at a wide range of universities might relate to. Future research should confirm that well-designed generic environments can achieve a similar impact across the wide diversity of college campuses and student populations across the country. Balancing realism and scalability will be critical to achieve a program appropriate for wide implementation.

Finally, consistent with research on the psychological impact of active shooter drills [57-59], implementation of VR-based training must be trauma-informed and include appropriate content warnings and opt-out options to minimize potential distress among users.

### Study Limitations

Several limitations of this study should be acknowledged. First, the study was conducted with a convenience sample of undergraduate students from a single large public university. Eligibility was limited to students aged 18-25 years, capturing prototypical undergraduate students but excluding perspectives from nontraditional or graduate students, as well as other campus community members such as faculty and staff. Given that just 2 focus groups were conducted, our findings may limit generalizability to other institutional types and student populations. Second, findings are based on self-reported perceptions of usability and acceptability rather than objective measures of learning, behavioral intention, or performance. Designed as a usability and feasibility study, it did not assess behavioral outcomes such as threat recognition, reporting behaviors, or simulated response effectiveness. Future research should incorporate those measures. Third, the Safe@Campus prototype included only 1 classroom-based scenario. Additional scenarios, such as outdoor spaces, residence halls, student unions, and dining facilities, will be incorporated into the final design of the program and may yield different usability considerations. Fourth, although stakeholder input informed development, broader multisite validation is needed to assess scalability and implementation feasibility across diverse campus infrastructures. Finally, immersive simulations addressing violence may evoke emotional distress. Although safeguards were in place, future research should rigorously assess psychological impact and incorporate trauma-informed best practices consistent with the emergency preparedness literature [57-59].

### Conclusions and Future Directions

This study provides foundational evidence supporting the feasibility and acceptability of Safe@Campus, a VR-based campus shooting preparedness intervention tailored to university settings. Findings also suggest that immersive technologies may bridge the gap between passive awareness and actionable preparedness for firearm injury prevention in university settings.

As firearm-related violence continues to affect university communities [2-4], institutions face increasing demand for effective, developmentally appropriate training. Safe@Campus represents a shift from passive informational approaches toward interactive, skill-based learning grounded in behavioral theory and public health principles. By allowing students to rehearse rapid decision-making in realistic campus environments, VR may enhance situational awareness, perceived behavioral control, and preparedness intentions consistent with TPB constructs [43,44].

Future research should continue to develop Safe@Campus and then evaluate its effectiveness through rigorous trials, including randomized clinical trials, which assess knowledge retention, self-efficacy, behavioral intentions, threat reporting, and simulated response performance. They should also rigorously assess the mental health aspects of exposure to the program. Longitudinal studies will be necessary to determine sustained impact. Expanding scenario diversity, increasing interactivity, and integrating prevention-focused modules aligned with threat assessment research will further strengthen ecological validity. If effective at scale, Safe@Campus may provide a scalable model for experiential firearm injury prevention in university settings, contributing to comprehensive campus safety and broader public health strategies to reduce violence-related harm.

## Data Availability

The datasets used and/or analyzed during this study are available from the corresponding author, JY, upon reasonable request. JY had full access to all the data in the study and takes responsibility for the integrity of the data and the accuracy of the data analysis. JY will be responsible for providing access to research data requested by third parties as freely and timely as possible unless a legal obligation restricts access to the data (eg, nondisclosure agreement), intellectual property protection, ethical approval requirements, ethical or security reasons, or other legitimate reasons.

## Abbreviations

OSSC: Ohio School Safety Center
OSU: The Ohio State University
TPB: theory of planned behavior
VR: virtual reality
